# Development of a culturally tailored medical nutrition therapy to improve dietary adherence in type 2 diabetes in Benin: an ORBIT model-based protocol

**DOI:** 10.3389/fnut.2026.1688980

**Published:** 2026-03-06

**Authors:** Halimatou Alaofè, Abidemi Okechukwu, Waliou Amoussa Hounkpatin, Adaeze Oguegbu, Kelly Jackson, Edward John Bedrick, John Ehiri

**Affiliations:** 1 Department of Health Promotion Sciences, Mel and Enid Zuckerman College of Public Health, University of Arizona, Tucson, AZ, United States; 2 School of Nutrition and Food Science and Technology, Faculty of Agricultural Sciences of the University of Abomey-Calavi (FSA-UAC) Campus d' Abomey-Calavi, Calavi, Benin; 3 Division of Public Health Practice, Policy and Translational Research, Mel and Enid Zuckerman College of Public Health, University of Arizona, Tucson, AZ, United States; 4 School of Nutritional Sciences and Wellness, College of Agriculture, Life and Environmental Sciences, University of Arizona, Tucson, AZ, United States; 5 Department of Epidemiology and Biostatistics, Mel and Enid Zuckerman College of Public Health, University of Arizona, Tucson, AZ, United States

**Keywords:** Type 2 diabetes, medical nutrition therapy, dietary adherence, ORBIT model, cultural adaptation, food security, Benin

## Abstract

**Background:**

Dietitian-led medical nutrition therapy (MNT) is an effective and cost-efficient strategy for improving dietary adherence and glycemic control in adults with type 2 diabetes (T2D). However, culturally adapted MNT interventions that account for local food systems, food security, and sociocultural eating practices remain scarce in African contexts. This paper describes the development of *Objectif Santé Diabète Bénin* (OSanDiaBé), a culturally tailored, dietitian-led MNT intervention designed to improve dietary adherence and glycemic control among adults with T2D in Benin, West Africa, using the Obesity-Related Behavioral Intervention Trials (ORBIT) model.

**Methods:**

To design OSanDiaBé, we use a hybrid framework integrating the ORBIT model with ecological validity and cultural adaptation approaches. In Phase Ia (Define), we developed a theory-driven model to identify key behavioral targets and hypothesized pathways linking a culturally tailored MNT intervention to dietary adherence and glycemic control. Phase Ib (Refine) involved adapting and refining an MNT intervention that combines evidence-based menu plans grounded in the 4A food security framework with individual nutrition counseling and group diabetes education. Intervention refinement was informed by mixed-methods data collected from 512 adults with T2D, including quantitative assessments, focus group discussions, sensory evaluations, and a stakeholder workshop, to enhance feasibility, acceptability, and cultural relevance.

**Expected outcomes:**

Phase II will evaluate feasibility, acceptability, and preliminary signals of effectiveness, including dietary adherence and glycemic control (HbA1c), prior to planned Phase III efficacy and Phase IV effectiveness trials.

**Conclusions:**

OSanDiaBé offers a replicable framework for culturally tailoring MNT interventions in low-resource settings. By integrating food security, culturally relevant dietary guidance, and family-centered nutrition support, this approach has the potential to strengthen diabetes nutrition care and reduce inequities in access to effective MNT across African contexts.

## 1 Introduction

Over the past decade, the prevalence of type 2 diabetes (T2D) in Africa has surged by 129%, with an estimated 55 million adults currently affected (1, 2). Beyond its clinical consequences, T2D imposes substantial burdens on families, communities, and health systems, contributing to increased healthcare costs, reduced productivity, and adverse mental health outcomes (3–5). Addressing this burden requires effective diabetes management strategies that extend beyond pharmacological treatment and are responsive to local sociocultural, economic, and food system contexts.

Strong evidence demonstrates that adherence to nutrition therapy guidelines improves glycemic control, reduces cardiovascular risk factors, and lowers diabetes-related complications in adults with T2D (6, 7). However, sustained dietary adherence remains challenging, as management often requires long-term changes to established eating patterns, including increased consumption of whole grains, lean proteins, and healthy fats, alongside reduced intake of refined carbohydrates and sodium (8–12). These changes are particularly difficult to maintain in settings characterized by limited nutrition knowledge, constrained household resources, and competing social and occupational demands (13). Additional barriers include limited family support, cultural incongruence of prescribed diets, economic constraints on food access, and inconsistent or conflicting dietary advice from healthcare providers (9, 14–17). Together, these challenges highlight the need for simplified, culturally relevant dietary guidance that aligns with local food systems and household decision-making contexts.

Dietitian-led medical nutrition therapy (MNT) is increasingly recognized as an effective and cost-efficient strategy for improving dietary adherence, lowering hemoglobin A1c (HbA1c), and supporting weight management in adults with T2D (18, 19). As the dietetics profession expands in Africa (20), dietitian-led MNT offers an opportunity to deliver standardized, evidence-based nutrition care through individualized counseling and structured education. However, much of the existing evidence base originates from high-income Western contexts, and few interventions in African settings have been systematically developed using staged, theory-driven translational models. Moreover, diabetes self-management education programs in low-resource settings often face challenges related to feasibility, acceptability, and sustainability, reflecting socioeconomic constraints and limited cultural adaptation (21, 22). These gaps underscore the need for interventions that are both contextually grounded and scalable within routine health systems (23, 24).

The present project aims to develop a culturally tailored, dietitian-led MNT intervention for adults with T2D that targets dietary adherence as the primary behavioral outcome and glycemic control as the primary biomedical outcome. The intervention integrates menu plans based on the 4A framework of food security—adequacy, affordability, accessibility, and cultural acceptability (25)—with individual nutrition counseling and group diabetes education. Intervention development is guided by the Obesity-Related Behavioral Intervention Trials (ORBIT) model, a systematic framework for the staged development and testing of behavioral interventions prior to large-scale efficacy trials (26). The ORBIT model comprises sequential phases, including defining behavioral targets and hypothesized mechanisms (Phase Ia), refining intervention components through formative and pilot work (Phase Ib), evaluating feasibility and preliminary efficacy (Phase II), and assessing efficacy and effectiveness in subsequent trials (Phases III and IV). Its emphasis on theory-driven development, contextual adaptation, and progression criteria aligns closely with the implementation-oriented focus of nutrition and public health research in low-resource settings (9, 14–17, 26–31).

This paper describes the development of *Objectif Santé Diabète Bénin* (OSanDiaBé), a culturally tailored, dietitian-led MNT intervention designed to improve dietary adherence and glycemic control among adults with T2D in Benin, West Africa. We present the theoretical framework, the cultural adaptation and refinement process, and the planned feasibility and pilot testing phases. Consistent with ORBIT Phase Ia objectives, this study addresses the following research question: What behavioral targets, contextual factors, and intervention components are required to develop a culturally tailored, dietitian-led MNT program capable of improving dietary adherence and glycemic control among adults with T2D in Benin ?

## 2 Methods

**Figure 1:**
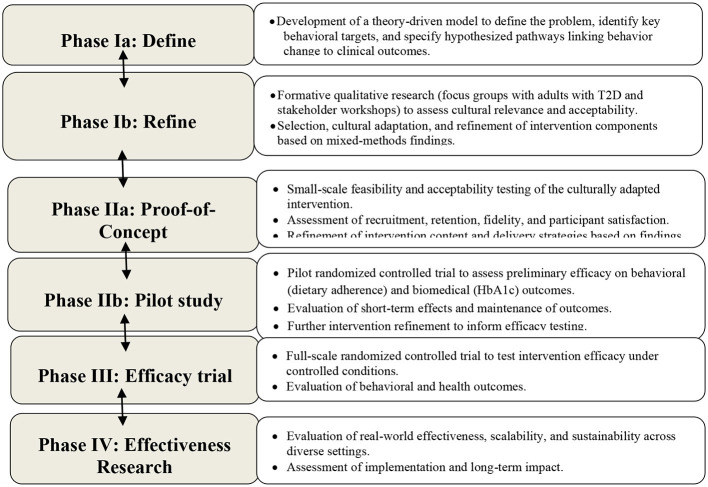
Summary of the ORBIT model [26, 27] applied to the development of ‘Objectif Santé Diabète Bénin' (OSanDiaBé) program for T2D in Africa, enabling progress and refinement based on new findings.

The development of OSanDiaBé was guided by the ORBIT model, a systematic and iterative framework designed to support behavioral intervention development prior to large-scale efficacy testing (26, 27). As illustrated in Figure 1 and described below, the ORBIT model emphasizes progressive refinement of intervention components to establish feasibility, acceptability, and cultural relevance before formal evaluation of efficacy and effectiveness. This framework is well suited to complex chronic disease management, such as T2D, where sustainable behavior change depends on addressing contextual, cultural, and psychosocial factors. Accordingly, analytical strategies across ORBIT phases were selected to align with the primary objective of intervention development. Descriptive and exploratory analyses were used to inform early-stage refinement, with increasing analytical complexity reserved for subsequent feasibility, pilot, efficacy, and effectiveness trials.

### 2.1 Phase Ia: define

**Figure 2:**
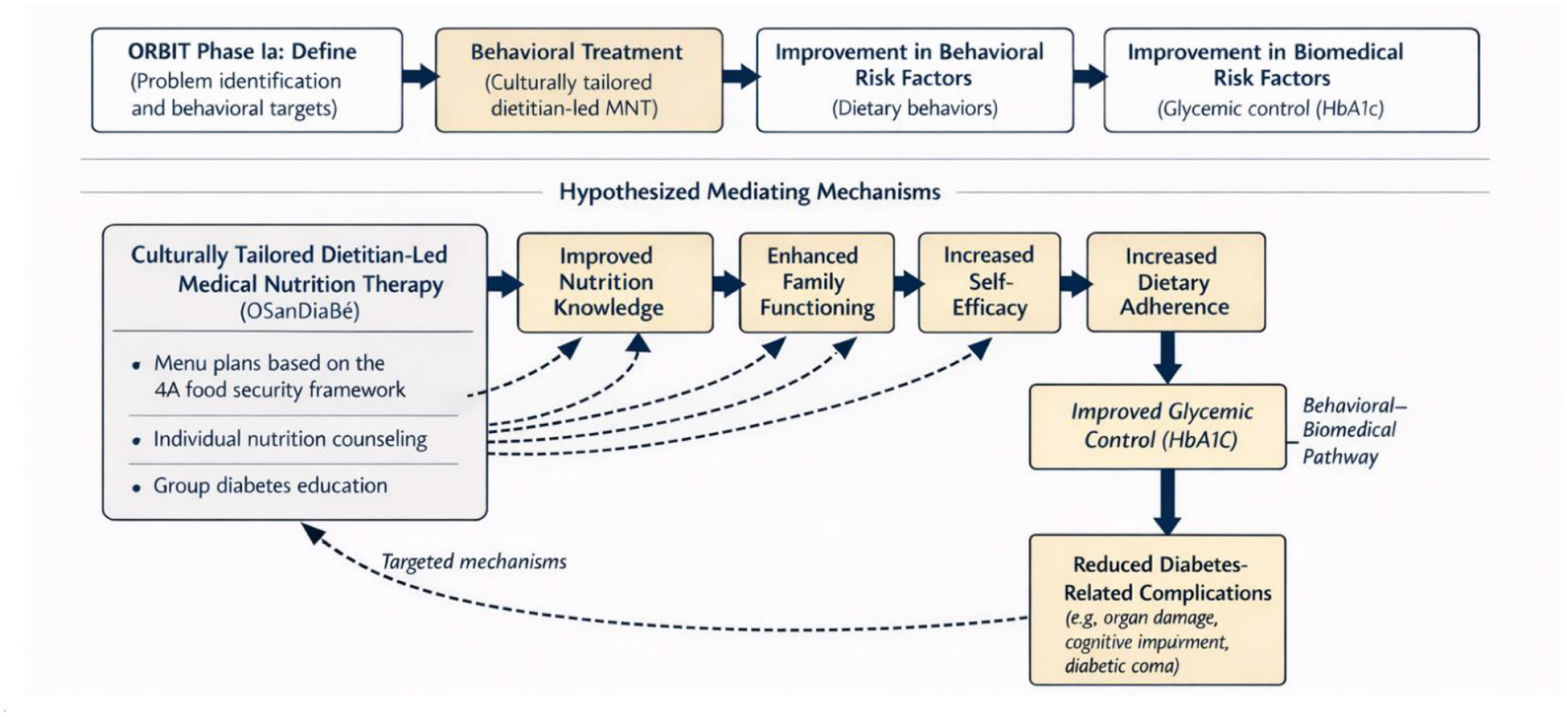
Application of the ORBIT model and theoretical framework guiding Phase Ia (Define) of the OSanDiaBé intervention.

In Phase Ia (Define), we developed a theory-driven model to articulate the behavioral problem, identify key intervention targets, and specify hypothesized pathways through which behavior change may influence clinical outcomes, consistent with the ORBIT framework. As illustrated in Figure 2, this model integrates the ORBIT Phase Ia process with a conceptual representation of how a culturally tailored, dietitian-led MNT intervention may improve dietary adherence and glycemic control among adults with T2D.

The model identifies dietary adherence as the primary behavioral target and addresses personal, familial, and environmental barriers commonly reported by adults with T2D in Benin. These barriers include limited nutrition knowledge, difficulties translating dietary recommendations into daily practice, cultural acceptability of prescribed diets, family dynamics, and economic constraints affecting food access. Identification of these barriers, informed by prior literature and formative engagement with local stakeholders, guided both the selection of intervention components and the specification of hypothesized mediating mechanisms.

We hypothesized that participation in the culturally tailored OSanDiaBé intervention—delivered through menu plans grounded in the 4A food security framework, individual nutrition counseling, and group diabetes education—would lead to improved dietary adherence and reductions in HbA1c. These effects were expected to be mediated by improvements in nutrition knowledge, family functioning, and self-efficacy, constructs supported by behavioral and social-cognitive theory. Improved dietary adherence was further hypothesized to result in better glycemic control and, over time, a reduced risk of diabetes-related complications, including organ damage and cognitive impairment. This theoretical model guided the development of intervention components and informed the selection of mediators examined in Phase Ib analyses.

### 2.2 Phase Ib: refine

In Phase Ib (Refine), we developed and refined the OSanDiaBé intervention components using an iterative, mixed-methods approach to enhance feasibility, acceptability, cultural relevance, and implementation fidelity, consistent with the ORBIT framework (Figure 1). This phase focused on refining both intervention content and delivery strategies through the systematic integration of quantitative and qualitative evidence. Quantitative data were used to characterize dietary adherence, family functioning, and glycemic control and to explore theory-informed relationships relevant to intervention refinement. Qualitative methods, including focus group discussions (FGDs), sensory evaluation, and stakeholder workshops, provided in-depth insights into cultural norms, perceived barriers and facilitators to dietary adherence, and the acceptability of proposed intervention components. Findings from these complementary data sources were triangulated to inform iterative modification of the intervention prior to feasibility and pilot testing.

#### 2.2.1 First stage of intervention manual development

The OSanDiaBé intervention manual was developed to operationalize the theoretical model defined in Phase Ia by translating identified behavioral targets and hypothesized mechanisms into culturally appropriate intervention components. A problem-formulation approach was employed to ensure that the intervention addressed the dietary, familial, and contextual barriers to adherence commonly reported by adults with T2D in Benin (32).

Intervention content and structure were informed by a theory of change, a comprehensive literature review, and formative qualitative research conducted with 32 adults with T2D, alongside consultations with 16 academic partners and 12 community partners in Benin (15–17). This formative work guided decisions regarding session content, delivery format, language and literacy level, recruitment materials, and participant engagement strategies.

Building on this foundation, evidence-based components were selected from established MNT and group education programs, including the Meta Salud Diabetes (MST) program (33) and the Pure Prairie Living Program (PPLP) (34, 35), both of which have demonstrated effectiveness in improving dietary understanding and addressing personal and environmental barriers to adherence. These components were systematically adapted using culturally adapted evidence-based practice approaches (36) and the ecological validity model (EVM) (37) to ensure alignment with local food systems, family structures, language, and health beliefs, as summarized in Table 1.

**Table 1:** Ecological validity model.

**Domain**	**Dimensions**	**Adaptations in the intervention**
**Language**	Culturally appropriate and culturally syntonic	Translation of materials into French (official language in Benin)
**Persons**	Attributes of persons involved and their relationship	Involvement of patients' partners to enhance support for healthy eating and lifestyles in households.
**Metaphors**	Culturally consonant sayings and stories.	Use of role plays, games, and storytelling to present the skills being taught in more relatable ways.
**Contents & concepts**	Concepts consonant with culture and context	Integration of culturally relevant MNT concepts and important lifestyle intervention topics.
**Goals**	Supportive of adaptive values of culture	Integration of patient behaviors with family communication skills alongside the MNT goals.
**Methods**	Strategies consonant with patients' culture	Use of visual aids and simple definitions to describe the content clearly.
**Context**	Consideration of contextual factors	Integration of menu plan based on the 4A framework and what-up messages for reminding, encouraging, motivating, and self-efficacy.

The intervention integrates individual nutrition counseling with group education to target multiple mechanisms of behavior change. Group sessions were designed to enhance family involvement, strengthen coping skills, and increase self-efficacy through guided practice, goal setting, and progress monitoring, while individual sessions supported personalized dietary planning and goal refinement. Consistent with MNT practice guidelines and prior behavioral interventions (38, 39), the program was structured to include 12 sessions, (four individual and eight group), considered sufficient to support knowledge acquisition, skill development, and the adoption of sustainable dietary behaviors (40–42).

#### 2.2.2 Intervention manual refinement

Refinement of the OSanDiaBé intervention manual employed a mixed-methods approach combining quantitative surveys, FGDs, sensory evaluation, and stakeholder workshops. This approach was designed to assess feasibility, acceptability, and cultural relevance of intervention components; validate proposed menu plans; and inform the integration of key actors, particularly family members and dietitians, into intervention delivery.

**In the quantitative stage**, participants were recruited from outpatient diabetes units of six secondary health centers in Benin (two public and four private). Eligible participants were adults aged 40–65 years with a documented diagnosis of T2D for at least 1 year, self-identified as Beninese, and willing to provide informed consent. Individuals with severe health conditions that could limit their ability to complete questionnaires or participate in anthropometric or biological assessments were excluded. The selected age range reflects the population most affected by T2D and its complications in Benin and sub-Saharan Africa and reduced heterogeneity in dietary behaviors and disease management experiences during intervention development. Requiring a minimum diabetes duration of 1 year ensured participants had prior exposure to diabetes self-management education and dietary recommendations, allowing meaningful assessment of dietary adherence, family functioning, and culturally embedded practices relevant to intervention refinement.

A total of 512 eligible participants were enrolled, consistent with recommendations for exploratory and formative research (43). Data collection included validated measures of dietary adherence, family functioning and support, cultural identity, and sociodemographic characteristics (age, sex, marital status, education, employment, income, ethnicity, and diabetes duration). Anthropometric measurements (height, weight, waist and hip circumference) and laboratory assessment of glycemic control (HbA1c) were also conducted.

**In the qualitative stage**, to complement quantitative findings, a random subsample of 94 participants was invited to participate in eight FGDs, consistent with qualitative research recommendations (44). Using a semi-structured guide, discussions explored understanding of food-based dietary guidelines, perceived barriers and facilitators to dietary adherence, acceptability and feasibility of proposed MNT components, perceptions of body size and obesity-related risks, and family dynamics related to food and diabetes management.

In addition, 30 participants (15 adults with T2D and 15 without T2D) were recruited to evaluate the acceptability of proposed dietary menus using sensory testing methods. Fourteen daily menus were developed using a linear goal programming model to optimize nutritional adequacy, affordability, accessibility, and cultural acceptability; detailed methods have been published previously (45). During sensory evaluation, trained participants rated packaged food samples using a five-point hedonic scale to assess overall acceptability (46).

Finally, a stakeholder workshop was conducted with 14 professionals involved in diabetes prevention and care, including dietitians, nutritionists, pharmacists, physical activity specialists, and endocrinologists, identified in collaboration with the Directorate of Health Services. The workshop focused on intervention delivery, recruitment strategies, cultural considerations, and implementation feasibility. All FGDs and workshops were digitally audio-recorded and transcribed for analysis.

#### 2.2.3 Quantitative analysis: phase Ib

All quantitative analyses were conducted using Stata (version 19; StataCorp LLC, College Station, TX, USA). Consistent with the objectives of ORBIT Phase Ib, analyses were designed to inform intervention refinement rather than to test intervention effectiveness.

Descriptive statistics, including means and standard deviations for continuous variables and frequencies and percentages for categorical variables, were used to summarize participant sociodemographic characteristics, dietary adherence, family functioning, and glycemic control. These analyses provided foundational information to guide refinement of intervention content, delivery strategies, and participant engagement approaches.

To examine hypothesized pathways specified in the Phase Ia theoretical model (Figure 2), mediation and moderation analyses were conducted using structural equation modeling (SEM) (47). These analyses assessed relationships among family functioning, dietary adherence, and glycemic control to identify psychosocial mechanisms relevant to intervention development and prioritization of intervention components. All SEM models were adjusted for age, sex, education level, diabetes duration, body mass index, health center, marital status, work status, and social support, variables known to influence dietary adherence and glycemic control, to reduce potential confounding (48, 49).

### 2.3 Phase IIa: proof-of-concept

Phase IIa will be conducted following completion of Phase Ib, as findings from the refinement phase are essential to determine the readiness of the OSanDiaBé intervention for feasibility testing. Consistent with the ORBIT framework, the primary objective of Phase IIa is to assess the feasibility and acceptability of the culturally tailored, dietitian-led MNT intervention using a mixed-methods design. We hypothesize that key feasibility indicators, including recruitment, retention, and session attendance, along with participant satisfaction and acceptability, will exceed 75%, indicating readiness for pilot testing in Phase IIb.

#### 2.3.1 Phase IIa sample

A single-arm feasibility design will be used to enroll 30 adults with T2D. Eligible participants will (1) self-identify as Beninese; (2) be aged 40–65 years; (3) have a clinical diagnosis of T2D for at least one year; (4) have an HbA1c level >7% at enrollment; (5) have a family member willing to participate or provide ongoing support; and (6) be willing to commit to the study protocol and attend scheduled sessions. Participants will be recruited from the pool of 512 individuals who participated in Phase Ib. The sample size of 30 was selected in accordance with recommendations for pre-pilot and feasibility studies (43). The intervention will include two individual sessions (45–60 min each) and eight group sessions (90–120 min each). Sessions will be delivered by six trained dietitians or nutritionists, with each group of 10 participants assigned two facilitators experienced in health promotion, trained in MNT, and committed to maintaining protocol fidelity.

#### 2.3.2 Phase IIa procedures

**Figure 3:**
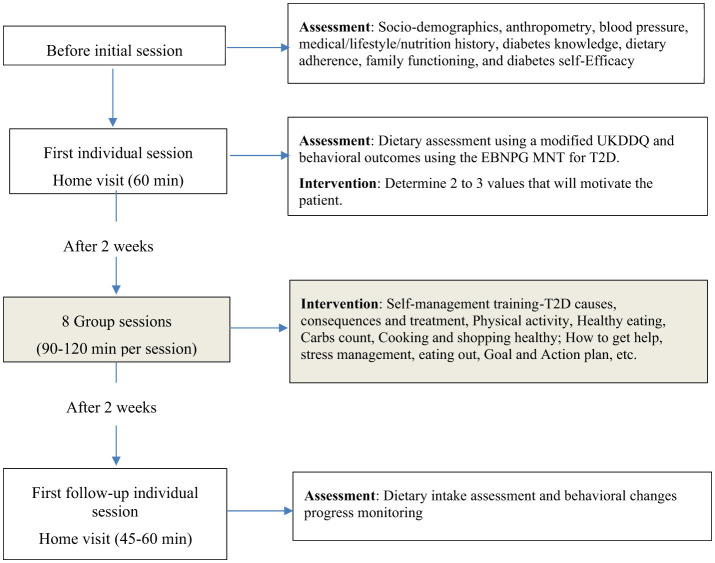
Schematic diagram of the feasibility study for the new cultural tailored medical nutrition therapy.

Participants will complete two individual sessions—one prior to and one following the group sessions—and eight group sessions delivered weekly or biweekly over a 6–8-week period (Figure 3). The initial individual session will last approximately 60 min, followed by a 45–60-minute follow-up session. Prior to the first visit, dietitians will collect relevant medical history and laboratory data. During the initial session, dietary intake will be assessed using the modified UK Diabetes and Diet Questionnaire (UKDDQ) to inform individualized nutrition prescriptions (50). Throughout the intervention, facilitators will monitor behavioral changes related to meal planning, food preparation, and physical activity, using a standardized adherence scale ranging from 1 (never) to 5 (consistently) (51). Family members will be invited to selected group sessions, including introductory and healthy-eating sessions, to promote a supportive home environment and reinforce family-centered dietary practices.

To support future replication and scalability, feasibility and acceptability outcomes will be evaluated using the RE-AIM (Reach, Effectiveness, Adoption, Implementation, and Maintenance) framework (52). Feasibility indicators will include recruitment strategies, session attendance, retention rates, and facilitator fidelity assessed through session observations. Acceptability will be measured using participant-reported perceptions of cultural relevance, perceived benefits, and overall satisfaction. Although Phase IIa is not designed to test efficacy, exploratory assessments of behavioral and biomedical outcomes will be conducted at baseline and 12 weeks post-intervention. Dietary adherence will be assessed using a multi-method approach combining the Perceived Dietary Adherence Questionnaire (PDAQ) adapted to the Benin Food Guide (53), dietary intake from the modified UKDDQ, and facilitator-rated behavioral monitoring (e.g., meal planning, food preparation, and physical activity practices) (51). Family functioning will be measured using the McMaster Family Assessment Device (54), diabetes knowledge using the ADKnowl questionnaire (55), and self-efficacy using the Diabetes Self-Efficacy Scale (DSES) (56). Anthropometric and clinical measures, including weight, height, waist circumference, blood pressure, and HbA1c—will also be collected with informed consent. Qualitative feedback from participants and facilitators will be analyzed to identify themes related to communication, comprehension, engagement, and perceived cultural relevance. Transportation incentives will be provided to facilitate participation in sessions and follow-up assessments.

#### 2.3.3 Quantitative analysis: phase IIa

Descriptive statistics will be used to summarize participant characteristics and feasibility and acceptability outcomes. Changes in exploratory behavioral and biomedical outcomes between baseline and post-intervention will be analyzed using paired-samples *t*-tests, Wilcoxon signed-rank tests, or McNemar's tests, as appropriate. Exploratory analyses will examine associations between dietary adherence scores and biomedical indicators (HbA1c and anthropometric measures) to assess convergent validity. All analyses will be conducted using Stata (version 19; StataCorp LLC, College Station, TX, USA), with statistical significance set at *P* < 0.05.

### 2.4 Phase IIb: pilot study

Phase IIb will be conducted following completion of Phase IIa to evaluate the preliminary efficacy of the OSanDiaBé intervention compared with usual care among adults with T2D. Consistent with the ORBIT framework, this phase is designed to generate initial estimates of intervention effects on key behavioral and biomedical outcomes and to assess readiness for a subsequent, fully powered efficacy trial. Usual care consists of standard clinical management, including routine blood glucose monitoring, medication adherence counseling, and general lifestyle advice delivered in routine practice (57). We hypothesize that participants receiving OSanDiaBé will demonstrate greater improvements in dietary adherence and greater reductions in HbA1c at 6 months compared with those receiving usual care. We further hypothesize that these improvements will be maintained at 12 months following the end of active intervention contact.

#### 2.4.1 Phase IIb sample

A total of 60 adults with T2D will be enrolled and randomized in a 1:1 ratio to either the intervention group (OSanDiaBé) or a wait-list control group receiving usual care. Randomization will be conducted after screening and informed consent using a computer-generated randomization sequence. To minimize potential bias, the principal investigator will be aware of group allocation, while physicians, data collectors, and laboratory personnel will remain blinded. Participants will be informed of their group assignment following randomization. The target sample size (*n* = 60; 30 per group) is consistent with recommendations for pilot randomized trials and is intended to support intervention refinement and effect size estimation for subsequent efficacy studies (58). Findings from Phase IIa will further inform final sample size considerations for later trials.

Participants will be recruited from six collaborating health centers using provider referrals, peer referrals, flyers, and word-of-mouth outreach. Eligibility criteria include: self-identification as Beninese; age 40–65 years; a clinical diagnosis of T2D for ≥1 year; uncontrolled diabetes (HbA1c >7%); availability of a supportive family member; and ability to provide written informed consent. Participants must be deemed suitable for group-based education and general dietary and lifestyle guidance by the referring healthcare provider. Exclusion criteria include pregnancy, recent major cardiovascular events (e.g., stroke or congestive heart failure), clinical conditions requiring specialized dietary management, or current participation in a similar nutrition program, to minimize contamination and ensure participant safety.

#### 2.4.2 Phase IIb procedures

The primary objective of Phase IIb is to evaluate the preliminary efficacy of OSanDiaBé in improving dietary adherence (primary behavioral outcome), and glycemic control (HbA1c) (primary biomedical outcome), as well as related T2D risk factors. Prior to study initiation, intervention materials will be finalized based on feedback from Phase IIa while preserving the core dietitian-led MNT framework. Facilitators will receive standardized training and lesson plans to ensure implementation fidelity.

As outlined in Table 2, participants in the intervention group will attend four individual sessions (45–60 min each) and eight group sessions (90–120 min each) during the first 6 months. The first two individual sessions will be delivered before and after the group sessions within the initial 3 months, followed by 2 monthly booster sessions between months 3 and 6. Group sessions will be delivered weekly or biweekly over an eight-week period. Intervention participants will receive the OSanDiaBé resource package, including a participant workbook, and an individualized Eating Plan based on the 4A food security framework. No intervention contact will occur between months 6 and 12. Participants in the wait-list control group will complete assessment visits at baseline, 3 months, 6 months, and 12 months and will receive usual care during the study period. Following completion of the 12-month assessment, they will be offered the OSanDiaBé intervention delivered by the same trained facilitators to ensure equitable access.

**Table 2:** Overview and timeline of the OSanDiaBé intervention and assessments (ORBIT Phase IIb).

**Study activities**	**Baseline (Month 0)**	**3 months (End of Core Delivery)**	**6 months (End of Active intervention)**	**12 months (Maintenance Follow-up)**
Enrollment, informed consent, and randomization	X			
Usual care (control group; standard clinical management throughout study)	X	X	X	X
Individual MNT sessions (*n* = 4; intervention group)	X	X	X	
Group MNT sessions (OSanDiaBé; *n* = 8; intervention group)		X (Months 0–3)		
Dietary assessments (e.g., dietary adherence, diet quality/intake screener)	X	X	X	X
Psychosocial assessments (family functioning, self-efficacy, diabetes knowledge, quality of life)	X	X	X	X
Anthropometric measurements (weight, height, BMI, waist circumference)	X	X	X	X
Metabolic assessments (HbA1c, blood pressure, lipid profile)	X	X	X	X

The active intervention phase spans Months 0–6. Core group-based OSanDiaBé sessions (n = 8) and the first two individual MNT sessions are delivered during Months 0–3. Two monthly booster individual MNT sessions are delivered during Months 3–6. The maintenance phase (Months 6–12) involves no intervention contact and includes follow-up assessments only.

Outcome assessments for both groups will be conducted at baseline and at 3, 6, and 12 months. Dietary adherence will be assessed using a multi-method approach combining the PDAQ adapted to the Benin Food Guide, dietary intake from the modified UKDDQ, and facilitator-based behavioral monitoring, consistent with the Phase IIb measurement strategy (50, 51, 53). Secondary outcomes will include: diabetes knowledge assessed using the ADKnowl questionnaire (55); family functioning measured with the 12-item General Functioning subscale of the Family Assessment Device (54); nutrition self-efficacy assessed using the DSES (56); and quality of life measured with the EuroQol EQ-5D (59). Anthropometric measures (weight, height, body mass index, waist circumference) and metabolic indicators (blood pressure, HbA1c, lipid profile) will be collected, with venous blood samples analyzed at an accredited hospital laboratory. In addition, focus group discussions with participants and facilitators will be conducted at 3, 6, and 12 months to capture experiential feedback related to intervention delivery, cultural relevance, and perceived impact, and to inform future program refinement.

#### 2.4.3 Quantitative analysis: phase IIb

All quantitative analyses will be conducted using Stata (version 19; StataCorp LLC, College Station, TX, USA). Normality of continuous variables will be assessed using the Kolmogorov–Smirnov and Shapiro–Wilk tests, with non-parametric methods applied as appropriate. Continuous variables will be summarized using means and standard deviations, and categorical variables using frequencies and percentages. Between-group comparisons will be conducted using Student's *t*-tests for continuous variables and chi-square tests for categorical variables. To examine changes in outcomes over time, repeated-measures analysis of variance (ANOVA) will be used to assess group-by-time interactions across baseline, 6-month, and 12-month assessments, with Bonferroni-adjusted *post hoc* comparisons. Models will adjust for baseline values of outcome measures. Statistical significance will be set at *P* < 0.05. Sensitivity analyses will be conducted using multiple imputation to address missing data and assess the robustness of findings.

#### 2.4.4 Qualitative analysis: phase Ib, IIa and Iib

Qualitative data from FDGs and interviews conducted across Phases Ib, Iia, and Iib will be audio-recorded, transcribed verbatim, and imported into Nvivo software (version 11.4) for analysis (60). A standardized transcription and data preparation protocol will be implemented to ensure accuracy and consistency (61, 62). Field notes will be integrated with transcript data, and all materials will be de-identified prior to analysis. To enhance analytic rigor, we will employ methodological triangulation by comparing qualitative findings with quantitative results across phases, and analyst triangulation through independent coding by multiple researchers (63). Data will be analyzed using a hybrid inductive–deductive content analysis approach, guided by the Phase Ia theoretical model and emergent themes. The analytic process will include preparation, coding, categorization, and reporting stages, conducted by two bilingual analysts to ensure linguistic and cultural accuracy (64, 65). Discrepancies in coding will be resolved through discussion and consensus.

## 3 Expected outcomes

### 3.1 Anticipated benefits and implications

Following completion of the intervention development, feasibility, and pilot phases, the OSanDiaBé program will be positioned for evaluation in a fully powered efficacy trial (ORBIT Phase III) and, subsequently, an effectiveness trial in real-world settings (ORBIT Phase IV). This systematic, theory-driven approach is expected to facilitate the development of culturally tailored, dietitian-led medical nutrition therapy (MNT) interventions while reducing the time, cost, and inefficiencies often associated with behavioral intervention development in low-resource settings. By emphasizing a clear chain of evidence from problem definition through pilot testing, the ORBIT framework provides a transparent and replicable pathway for progression from conceptualization to efficacy testing. The OSanDiaBé development process illustrates how culturally grounded dietary interventions—anchored in local food systems and family contexts—can be systematically designed, refined, and prepared for scale-up. Importantly, this framework is adaptable across settings and health conditions, offering methodological flexibility for the development of nutrition-based interventions for chronic disease management in Africa and comparable low-resource contexts (26).

### 3.2 Strengths and limitations of the study

This study has several notable strengths. First, use of the ORBIT model supports a structured yet flexible approach to intervention development, treating challenges encountered during early phases as opportunities for refinement rather than failure. Second, integration of mixed methods—including quantitative assessments, qualitative inquiry, and session observations—enhances cultural adaptation, contextual relevance, and implementation fidelity. Third, the OSanDiaBé intervention is explicitly grounded in the 4A food security framework, promoting dietary patterns that are nutritionally adequate, culturally acceptable, and feasible within local food environments in Benin. By emphasizing locally available foods, the program also supports short food supply chains, which may contribute to improved metabolic health (66).

The project further aligns with the expansion of the dietetics profession in Africa and with World Health Organization action plans aimed at reducing the burden of diabetes through capacity building and strengthened nutrition care (20, 67, 68). Nonetheless, several limitations warrant consideration. Participants were predominantly recruited from Cotonou in southern Benin, which may limit generalizability to other regions; future studies will include more geographically diverse samples. Restricting the age range to 40–65 years enhanced internal validity during intervention development but limits applicability to younger and older adults; subsequent trials will broaden eligibility criteria. Also, variability in diabetes duration among participants may influence experiences and outcomes; future analyses will stratify participants by disease stage to better account for these differences.

### 3.3 Possible pitfalls and alternative strategies

A key challenge is the recruitment and retention of participants with limited prior exposure to MNT research. While strong community partnerships and formative engagement support current recruitment strategies, alternative approaches—such as collaboration with religious institutions, community leaders, and local organizations—will be implemented if recruitment targets are not met. To promote retention, participants will receive transportation compensation, flexible scheduling, and culturally responsive counseling tailored to individual and family needs. For participants unable to attend sessions in person, home-based counseling will be offered to maintain engagement and intervention fidelity. If Phase IIa identifies low feasibility or acceptability, targeted mixed-methods analyses will be conducted to identify underlying barriers, and intervention components will be adapted accordingly before progression to subsequent phases. This iterative process, consistent with the ORBIT framework, ensures that the intervention is optimized for effectiveness, scalability, and sustainability in real-world settings.

## 4 Discussion

This study describes the development and planned evaluation of OSanDiaBé, the first culturally tailored, dietitian-led MNT intervention for adults with type 2 diabetes in Benin developed using the ORBIT model (26, 27). Methodological decisions were guided by the objective of designing a scalable and culturally responsive MNT intervention that addresses dietary adherence and glycemic control within the sociocultural and food system context of Benin. Integration of the ORBIT framework with mixed-methods and cultural adaptation approaches enabled systematic refinement of intervention components while maintaining alignment with local food systems, family structures, and health beliefs (26, 36, 37).

The emphasis on descriptive and exploratory analyses is consistent with the objectives of ORBIT Phase Ib, which prioritize understanding behavioral, familial, and contextual determinants to inform intervention refinement rather than formal hypothesis testing for effectiveness (26). Importantly, incorporation of mediation and moderation analyses extends beyond descriptive reporting to identify potential psychosocial mechanisms—such as family functioning and self-efficacy—through which culturally tailored MNT may influence dietary adherence and glycemic control (45–47). This theory-driven analytic approach strengthens the conceptual foundation of OSanDiaBé and informs targeted refinement of intervention components prior to feasibility and pilot testing.

Central to the OSanDiaBé development process is sustained engagement with patients and key stakeholders. Ongoing collaboration with adults with T2D, healthcare professionals, and community partners ensured that lived experiences and local perspectives informed intervention content, delivery, and adaptation (15–17). This participatory approach supports iterative refinement during ORBIT Phase IIa (proof-of-concept) and Phase IIb (pilot study) and positions the intervention for subsequent efficacy and effectiveness trials (26). Collectively, this project contributes to advancing culturally adapted, dietitian-led MNT interventions in Africa, strengthening nutrition care for adults with T2D, and addressing disparities in access to effective dietary support (20, 65, 66). By equipping dietitians with contextually appropriate tools and evidence-based frameworks, OSanDiaBé has the potential to enhance existing diabetes care pathways and promote equitable, scalable, and sustainable nutrition interventions in low-resource settings.

## Data Availability

The original contributions presented in the study are included in the article/supplementary material, further inquiries can be directed to the corresponding author.
